# Transcranial alternating current stimulation over left DLPFC modulates feedback processing: a simultaneous tACS-fMRI study

**DOI:** 10.1038/s41398-026-03942-6

**Published:** 2026-03-18

**Authors:** Ranjan Debnath, Eva Lenz, Johannes Tobelander, Paula Schweppe, Vincent Renner, Christoph Mulert

**Affiliations:** 1 Centre for Psychiatry and Psychotherapy, Justus-Liebig University Giessen, Giessen, Germany; 2 Centre for Mind, Brain and Behavior (CMBB), University of Marburg and Justus-Liebig University Giessen, Marburg, Germany

**Keywords:** Neuroscience, Learning and memory, Human behaviour

## Abstract

Studies have shown that reward-related feedback is processed by distinct brain networks. Neuronal oscillations, particularly theta and beta rhythms, play a key role in facilitating communications within these networks. This study investigated how modulating brain oscillations at theta and beta frequencies using transcranial alternating current stimulation (tACS) influences activation in brain regions involved in feedback processing. In three separate sessions, 28 healthy participants received either theta (5 Hz), beta (25 Hz), or sham tACS over the left dorsolateral prefrontal cortex (DLPFC) while performing a gambling task. fMRI data were recorded simultaneously with tACS to measure BOLD activations associated with gain and loss feedback. Results showed that theta stimulation enhanced activity in brain regions related to sensory processing, error monitoring, cognitive control, and emotion regulation during loss feedback. On the other hand, beta stimulation modulated activation in areas associated with reward sensitivity and emotional processing during gain feedback. These findings demonstrate the distinct roles of theta and beta oscillations in negative and positive feedback processing.

## Introduction

Evaluating the outcomes of our actions is fundamental to effective decision-making and adaptive behavior. The evaluation process relies on feedback from performed actions to assess outcomes, adjust strategies, and optimize future responses. In humans, feedback is broadly categorized into error-based (negative or loss) and reward-based (positive or gain) feedback, each contributing to learning and adaptive behavior [1, 2]. Feedback is primarily processed by overlapping brain regions, particularly those involved in reward and error-monitoring systems, which interact with cognitive and emotional regions to encode reward-related information, evaluate feedback, and guide adaptive decision-making [3–5].

The neural underpinnings of feedback processing are widely studied through a well-established event-related potential component known as the feedback-related negativity (FRN) [6]. FRN is a negative deflection occurring approximately 250–300 ms after negative feedback over frontocentral areas and is thought to originate in the anterior cingulate cortex (ACC) and medial frontal cortex [7, 8]. Neuroimaging studies further reveal that feedback processing relies on an extensive network, including the ventral tegmental area, striatum, amygdala, insula, ACC, and prefrontal cortices [3–5]. However, the mechanisms by which these regions integrate information and interact remain poorly understood. EEG research suggests that neuronal oscillations may be a key mechanism for coordinating activity across the feedback network [9]. EEG studies have extensively investigated theta and beta rhythms as markers of oscillatory brain activity related to feedback processing. Negative feedback is primarily associated with increased theta power and phase synchronization [10–12]. In contrast, positive feedback elicits increased beta power [13–16]. Moreover, theta oscillations are predominantly modulated by feedback valence [17], whereas beta oscillations are driven by feedback related to the probability and magnitude of reward [10, 14]. These findings suggest that distinct oscillatory mechanisms underlie the processing of positive and negative feedback [18].

Given the role of oscillations in coordinating feedback processing, modulating them in specific brain regions may provide critical insights into their functional role. Non-invasive brain stimulation techniques offer a powerful tool for this purpose. For instance, Nejati et al. [19] found that transcranial direct current stimulation over the ventromedial and dorsolateral prefrontal cortex (DLPFC) reduced impulsive decision-making and increased the preference for delayed rewards in children with ADHD. Similarly, Yaple et al. [20] demonstrated that 20 Hz transcranial alternating current stimulation (tACS) over the left prefrontal cortex increased risk-taking behavior, highlighting the role of beta oscillations in reward-based decision-making. However, while previous studies have explored the impact of oscillatory modulation on reward-based decision-making, the effects of targeted oscillatory stimulation on reward-related feedback processing (e.g., loss and gain feedback) remain largely unexplored. To address this gap, we used tACS combined with fMRI to investigate how modulating theta and beta oscillations influences feedback processing in healthy individuals. tACS is particularly well-suited for this purpose, as it is thought to interact with ongoing neural oscillations at stimulation frequency, thereby modulating neural excitability and cognitive processes [21–23]. However, recent evidence shows that such effects can depend on phase relationships and task context, which may lead to diverse outcomes [24, 25]. Despite its potential, research using tACS to probe the neuronal mechanisms of feedback processing remains scarce.

The present study examined how theta (5 Hz) and beta (25 Hz) tACS over the left DLPFC would affect neural activation during feedback processing. Previous research suggests that the DLPFC plays a pivotal role in feedback processing [18, 26–28]. Our previous work demonstrated that negative feedback increased theta power and was associated with the ACC and DLPFC activations [18]. In contrast, positive feedback increased beta power and engaged core reward network regions [18]. A follow-up study further demonstrated that theta responses were more strongly linked to left DLPFC activation during loss feedback [28]. HajiHosseini and Holroyd [26] localized the increase in beta power following positive feedback to the right DLPFC. Given the DLPFC’s involvement in feedback processing, modulating its oscillatory activity with tACS allows us to investigate its functional role and assess how stimulation may influence the broader feedback network.

Beyond its theoretical contributions, the present study also holds potential clinical implications. Increasing evidence suggests that deficits in feedback processing contribute to various neuropsychiatric conditions such as impulsivity, psychosis, depression, mood disorders, substance use disorders, borderline personality disorder (BPD), and schizophrenia [29–37]. For instance, our previous work demonstrated that individuals with BPD [29] and schizophrenia [32] exhibited reduced theta activity in response to negative feedback and decreased beta activity to positive feedback, which indicates impaired feedback processing. Similar deficits are observed in depression and related mood disorders, where hypoactivity in reward-related circuits correlates with anhedonia and impaired decision-making [34, 36]. In contrast, heightened reward sensitivity and diminished punishment responses exacerbate impulsive behaviors in substance use disorders [30]. Of particular relevance to the current study, clinical findings highlight DLPFC as a key region involved in feedback processing, as individuals with BPD exhibit reduced theta power and diminished DLPFC activation in response to negative feedback [28].

In this study, we employed a well-established gambling paradigm and applied theta and beta tACS over left DLPFC while participants underwent fMRI. Based on the previous findings, we hypothesized that theta tACS would modulate brain activity primarily during negative feedback, whereas beta tACS would do so during positive feedback. Specifically, we expected theta tACS to enhance activation in the error monitoring and cognitive control regions, including the ACC and DLPFC, in response to negative feedback compared to sham stimulation. In contrast, beta tACS was expected to increase activation in reward-related regions such as the striatum and affective areas following positive feedback. Additionally, we explored potential relationships between impulsive personality traits and stimulation-induced brain activation during feedback processing. Combining tACS with fMRI provides a novel approach to investigating how oscillatory modulation influences feedback processing at the network level. Previous work has demonstrated that tACS effects on the BOLD signal are frequency- and task-dependent, with the strongest effects often observed in distal brain regions, suggesting network-level modulation [38]. Our findings may provide new insights into the neural mechanisms underlying feedback processing and have implications for neuropsychiatric conditions characterized by deficits in feedback processing.

## Materials and methods

### Participants

We initially recruited 28 healthy participants for this study. The inclusion criteria required participants to be healthy adults with normal or corrected-to-normal vision, no known history of neurological or psychiatric disorders, and eligibility for MRI and tACS procedures (e.g., no metallic implants). Exclusion criteria included technical problems during data acquisition and noncompliance. Accordingly, four participants were excluded: three due to technical issues and one for falling asleep during an MRI session. Consequently, the final sample consisted of 24 healthy volunteers (17 female) aged between 20 and 55 years (mean = 24.83, std = 6.99). Each participant performed a gambling task in the MR scanner in three separate sessions, with a time interval of 7 (±2) days between sessions. During the fMRI scans, participants received tACS in one of three conditions: beta, theta, or sham stimulation. The order of the tACS conditions was counterbalanced across participants to minimize sequence effects. All participants had normal or corrected-to-normal vision and reported no history of neurological or psychiatric disorders. The study was conducted following the Declaration of Helsinki and was approved by the Ethics Committee of Justus Liebig University, Giessen. All participants provided written informed consent before participating in the study.

### Psychometric data

At the first assessment, sociodemographic and health-related data were collected, and participants completed psychometric questionnaires. Trait impulsivity was measured using the Barratt Impulsiveness Scale-11 (BIS-11), a 30-item self-report measure that assesses three domains of impulsivity: attentional, motor, and non-planning impulsivity [39].

### Gambling task

**Fig. 1:**
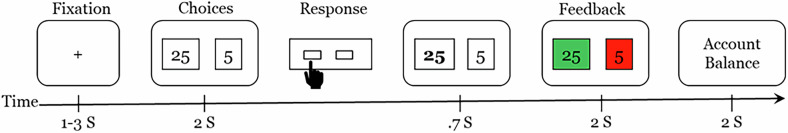
A schematic illustration of the gambling task used in this study.

The participants performed a computerized two-choice gambling task adapted from Gehring & Willoughby [7], which has been employed in previous studies [17, 18, 29, 40, 41]. The experiment used Presentation software for stimulus presentation. During fMRI, participants viewed stimuli on a screen through a mirror mounted on the MR head coil. Each trial started with a fixation cross displayed for 1 to 3 s (jittered by 500 ms), followed by two numbers (5 and 25) presented in the middle of a computer screen as two possible displays: either [25] [5] or [5] [25] in a randomized order (Fig. 1). Participants were required to select one of the two numbers by pressing a button within 2 s. Two seconds after stimulus onset, the font weight of the selected number was set to bold. After a further delay of 700 ms, one of the two numbers randomly turned green and the other red as feedback. This color change represented the feedback that indicated whether the participant had gained or lost. If the selected number turned green, the participant gained the corresponding points, while the red color indicated a respective loss of points. The feedback was displayed for 2 s, followed by a display of the current account balance for 2 s (Fig. 1). If no response was made within 2 s, the trial was terminated, and the next trial began.

The paradigm consisted of a short practice block and two experimental blocks, each comprising 100 trials. A 28-second inter-block interval separated the two blocks. Participants were given standardized instructions to freely choose one of the two presented numbers (5 or 25) in each trial and to earn as many points as possible in each block. Thus, they were not required to choose 5 and 25 in equal proportions. Participants started each block with 1000 points in their account. The instruction stated that they would receive a fixed payment for study participation, plus an additional amount equivalent to the total points gained after deducting an initial 1000 credit points (calculated in cents). They were assured that a negative balance would not result in any financial loss. Gain and loss outcomes occurred with equal probability (50%) for each number. Trial sequences were pseudo-randomized so that every combination of number (5 or 25 points) and feedback (gain or loss) occurred equally often, and no more than two identical outcomes occurred consecutively. To avoid confounds between outcome valence (gain vs. loss) and decision correctness, the two numbers were always changed to different colors. The entire task lasted for approximately 45 min. Participants received compensation (€10 per hour) for their participation.

### fMRI data acquisition

Imaging was performed using a 3-Tesla MR scanner (Siemens Prisma, Munich, Germany) equipped with a 64-channel head coil. Field map sequences were applied before the EPI sequence to control for magnetic field inhomogeneities. Functional blood-oxygen-level-dependent (BOLD) imaging was performed using a standard gradient-echo planar imaging (EPI) T2*-sensitive sequence, which acquired 42 slices. The EPI acquisition parameters were: TR = 2 s, TE = 30 ms, FOV = 220 mm, interleaved slice acquisition, echo spacing = 0.6 ms, and slice thickness = 3 mm. After removing the tACS setup, a high-resolution T1-weighted anatomical image (MPRAGE) was acquired for each participant with a voxel size of 0.9 × 0.9 × 0.9 mm.

### tACS

tACS was delivered using an MRI-compatible 1-channel neurostimulator system (DC-Stimulator plus by neuroConn GmbH, Ilmenau, Germany). The setup followed the operating instructions provided by neuroConn GmbH (DC-Stimulator plus, version 4.1.0). A cable connected the outer box, placed outside the Faraday cage, to the inner box located in the MRI room via the waveguide. The MR-compatible stimulation electrode cables were attached to the inner box. Round rubber electrodes with a diameter of 2 cm were used, and an EEG cap was used for electrode placement. The stimulation electrodes were positioned at F5 and FT9 locations according to the 10–20 system. A 5 mm layer of electrode paste (Ten20 Paste, Weaver and Company, Aurora, USA) was applied to the electrodes, and SuperVisc electrode gel (EASYCAP GmbH, Wörthsee, Germany) was injected to improve conductivity. Impedance was checked outside the scanner and maintained below 25 kΩ.

During the gambling task performed inside the fMRI scanner, participants received beta, theta, or sham stimulation for approximately 40 min, with a 10-second ramp-up and 10-second ramp-down period. The stimulation was delivered at a peak-to-peak intensity of 1.5 mA (±0.75 mA zero-to-peak; total charge Q = 3.6 Coulombs). Beta stimulation was applied as a sinusoidal waveform at 25 Hz, and theta stimulation at 5 Hz, both with no DC offset. Stimulation commenced with the onset of the EPI sequence and lasted until its conclusion. Sham stimulation was delivered at the start of the EPI sequence and consisted of a 10-s ramp-up, 60-s stimulation at 1 Hz (±0.75 mA zero-to-peak), and a 30-s ramp-down, after which the current was turned off.

**Fig. 2:**
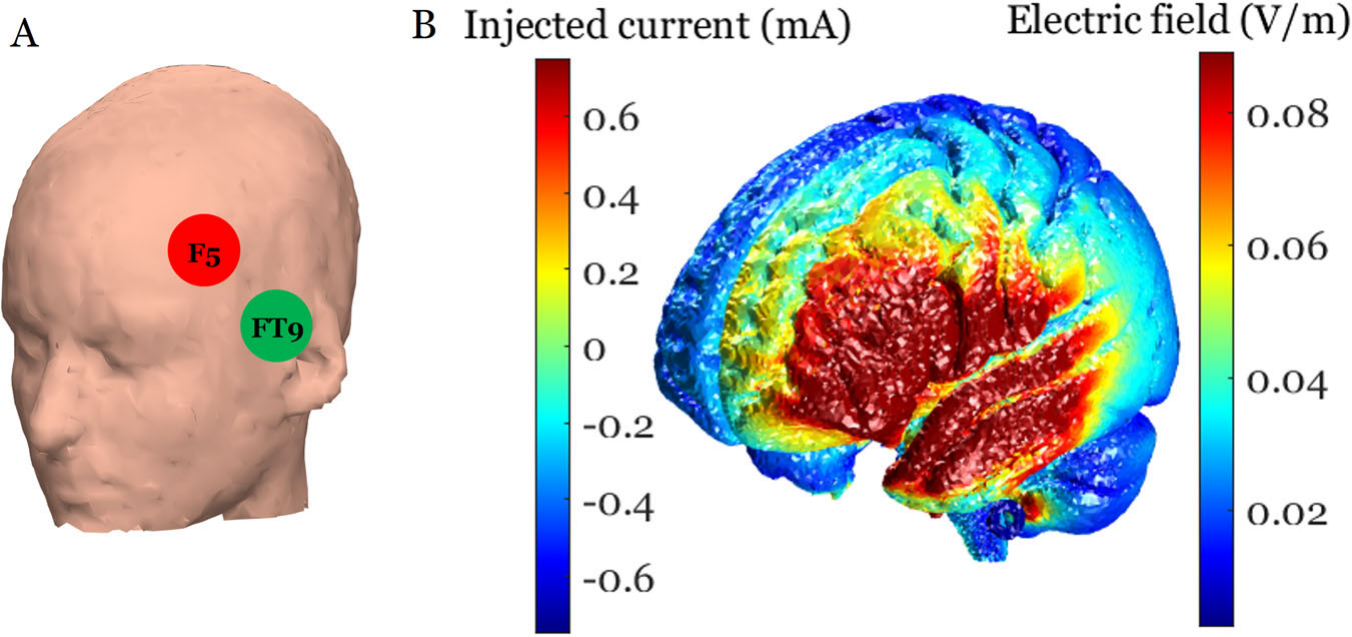
Electrode montages and corresponding electric field distributions for the tACS protocol. **A** Electrode configuration used for stimulation. **B** Simulated current flow and resulting electric field distribution generated using the ROAST toolbox. The simulation indicates that the tACS protocol effectively concentrated the electric field within the left DLPFC, confirming that the chosen montage effectively targets the intended stimulation target.

The target of stimulation was the left DLPFC at MNI coordinates [–40, 40, 20]. Stimulation was delivered using a two-electrode montage with electrodes positioned at F5 (stimulating) and FT9 (return) according to the international 10–20 EEG system. To verify that this montage effectively targeted the left DLPFC, electric field distributions were modeled using the ROAST toolbox [42]. ROAST constructs a realistic finite-element head model from structural MRI data or a standard anatomical template and estimates the resulting intracranial electric field distribution for a specified electrode montage. We applied the ROAST pipeline using the ICBM MNI152 T1-weighted template (1 mm isotropic resolution), and stimulation targets were defined using MNI coordinates. The modeled electric field was subsequently scaled to correspond to the experimentally applied stimulation intensity (±0.75 mA zero-to-peak). Simulation results confirmed that the maximal electric field was localized to the left DLPFC, indicating that the chosen montage effectively targeted the intended region (Fig. 2). The stimulation parameters complied with established safety guidelines for transcranial electrical stimulation in humans [43, 44].

### fMRI data analysis

The fMRI data were processed using SPM12 (Statistical Parametric Mapping Software; Wellcome Centre for Human Neuroimaging, University College, London, UK) running under MATLAB R2023a. The first five volumes from each EPI recording session were discarded to allow the magnetic field to reach a steady state. Preprocessing included spatial realignment with unwarping, co-registration of the first functional image to the anatomical MPRAGE image, normalization to the MNI template using segmentation, and spatial smoothing with a 6 mm full-width at half-maximum (FWHM) Gaussian kernel. At the first level, general linear models were fitted for each participant to model the individual BOLD signal changes. For each of the three tACS conditions (beta, theta, sham), the following conditions were modelled as separate regressors and convolved with the canonical hemodynamic response function: (a) initial stimulus presentation, (b) motor response, (c) anticipation phase, (d) feedback conditions (large gain, large loss, small gain, small loss), (e) account balance presentation, and (f) instruction presentation. Six motion parameters were included as regressors of no interest.

Contrasts of interest were defined at the first level and analysed using one-sample or two-sample t-tests at the second level, as appropriate. To confirm that the experimental paradigm elicited the expected effect, we first examined the contrast gain > loss under sham stimulation. To assess the effects of beta stimulation during gain feedback, we analysed the contrast gain(beta) > gain(sham). Similarly, to investigate the effects of theta stimulation, we analysed the contrast gain(theta) > gain(sham). For loss feedback, we examined the contrast loss(beta) > loss(sham) and loss(theta) > loss(sham). Finally, to directly compare the two active stimulation conditions, we tested the contrasts gain(beta) > gain (theta) and gain(theta) > gain(beta) for gain feedback. Similarly, we tested the contrast loss(beta) > loss(theta) and loss(theta) > loss(beta) for loss feedback. Statistical significance was assessed using a voxel-wise uncorrected threshold of p < 0.001, followed by a cluster extent threshold of k ≥ 110 voxels. Effects were considered significant if clusters exceeded the voxel-wise threshold and survived false discovery rate (FDR) correction (p < 0.05) at the cluster level.

For region of interest (ROI) analyses, voxel-level statistics were examined using a significance threshold of p < 0.05, corrected for multiple comparisons using family-wise error (FWE) correction. ROIs were selected based on previous studies using the same gambling task, focusing on brain areas consistently implicated in reward- and loss-related processing [18, 28, 45]. ROI masks were derived from the Automated Anatomical Labeling (AAL) atlas [46]. For gain-related contrasts, the following ROIs were selected using AAL MNI V4 masks: putamen, caudate nucleus, hippocampus, amygdala, and parahippocampal gyrus. The nucleus accumbens was defined using the AAL MNI V3 mask. To identify activations in the DLPFC, Brodmann area (BA) 9 and BA 46 masks were applied; for the ventrolateral prefrontal cortex (vlPFC), BA 10 and BA 11 masks were used. For loss-related contrasts, the ROIs included the anterior cingulate cortex, putamen, caudate nucleus, amygdala, and thalamus (all defined using AAL MNI V4 masks). DLPFC and vlPFC were again defined using the same Brodmann area masks as in gain-related contrasts.

### Association between brain activation and impulsive personality

We examined the relationship between tACS-induced brain activation and impulsive personality traits. Bold activations of regions of interest (ROIs) that emerged as significant in the above analyses were correlated with impulsivity data. Using MarsBar (marsbar.sourceforge.net), spherical ROIs with a 5 mm radius were constructed around the peak voxel of each significant cluster. The mean beta value within these ROIs was extracted as the measure of activation. This method enables the investigation of how individual differences in brain activity relate to impulsive personality traits. We conducted Spearman’s rank correlation to assess the relationship between BOLD activation and impulsivity measure, as this method is robust to potential outliers. No correction for multiple comparisons was applied, as these analyses were exploratory in nature.

## Results

**Fig. 3:**
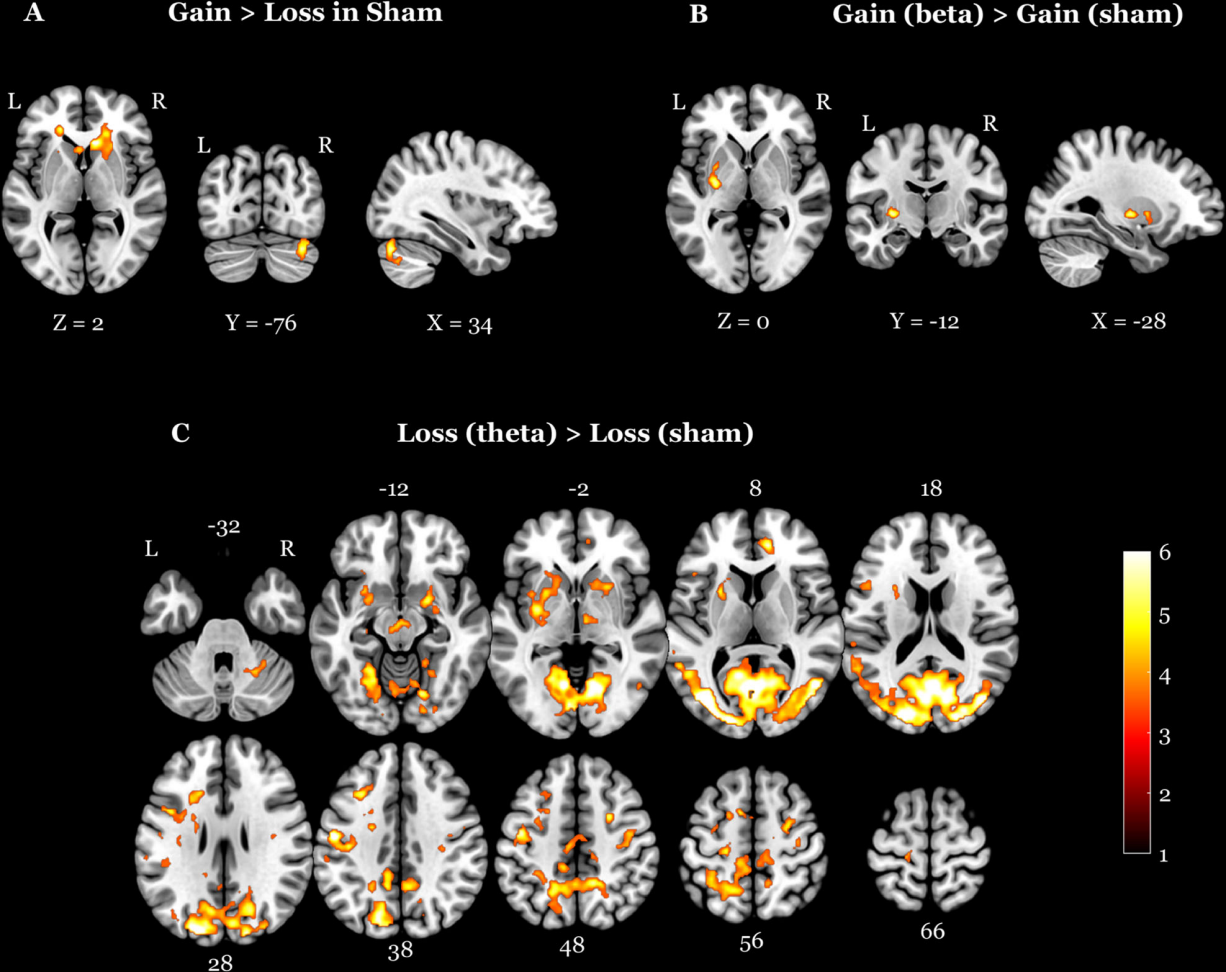
BOLD responses during gain and loss feedback. **A** Areas showing greater BOLD response for gain vs loss feedback at the cluster level for the sham stimulation. **B** Areas showing activations during gain feedback processing for the contrast beta sham. **C** Areas showing activations during loss feedback processing for the contrast theta sham. The opposite contrasts did not yield significant results in any of the above cases. BOLD blood-oxygen-level dependent.

For the contrast gain > loss under sham stimulation, significant activations were observed on the whole brain level and in pre-defined ROI (Fig. 3a and Table 1). Our primary focus was on the contrasts involving brain stimulation. The contrast gain(beta) > gain(sham) under beta stimulation revealed significant activations in bilateral putamen (L putamen *p* = 0.002, x = −28, y = −12, z = 0, *T* = 5.82; R putamen *p* = 0.01, x = 30, y = 2, z = 4, *T* = 5.06) and left amygdala (*p* = 0.048, x = −26, y = −2, z = −12, *T* = 3.54) (Fig. 3b and Table 2). The contrast gain(theta) > gain(sham) under theta stimulation showed significant activations in left DLPFC (*p* = 0.008, x = 52, y = 10, z = 3, *T* = 5.23) and left putamen (*p* = 0.006, x = −22, y = 4, z = 6, *T* = 5.22) (Table 3). The loss(beta) > loss(sham) comparison showed significant activation only in the ROI analysis of the left putamen (*p* = 0.011, x = −28, y = −12, z = −2, *T* = 4.98). In the loss condition, theta stimulation considerably impacted the BOLD activation. The contrast loss(theta) > loss(sham) revealed increased activation in various regions of the sensory, affective, and cognitive processes (Fig. 3C and Table 4).

**Table 1:** Localization and statistics of the voxels for gain > loss under sham.

brain structure	*P*	*x*	*y*	*z*	*T/K*
WB cluster level					
R nucleus caudate	<0.001	10	24	2	1021
R occipital fusiform gyrus	0.007	34	−76	28	206
ROI					
R amygdala	0.032	22	−6	−12	3.68
L caudate	0.001	−12	20	−4	6.06
R caudate	0.001	12	24	2	6.19
R dACC (BA24)	0.046	4	−4	28	4.06
L hippocampus	0.047	−12	−36	4	4.11
L nucleus accumbens	0.002	−8	16	−2	3.96
R nucleus accumbens	<0.001	10	10	4	4.14
L putamen	0.024	−20	4	−8	4.44
R putamen	0.006	16	12	−4	5.12
L thalamus	0.024	−12	−34	4	4.45

WB threshold = *T* ≥ 6.43. All coordinates (x, y, z) are given in MNI space.

*WB* whole brain, L left, *R* right.

**Table 2:** Localization and statistics of the voxels for tACS(beta/theta) > sham in the gain condition.

gain(beta) > gain(sham)					
brain structure	*P*	*x*	*y*	*z*	*T/K*
WB cluster level					
L putamen	0.010	−28	−12	0	119
ROI					
L amygdala	0.048	−26	−2	−12	3.54
L putamen	0.002	−28	−12	0	5.82
R putamen	0.010	30	2	4	5.06
**gain(theta) > gain(sham)**					
ROI					
L DLPFC (BA9)	0.008	52	10	34	5.23
L putamen	0.006	−22	4	6	5.22

All coordinates (x, y, z) are given in MNI space.

*WB* whole brain, *L* left, *R* right.

**Table 3:** Localization and statistics of the voxels for tACS(beta/theta) > sham in the loss condition.

loss(beta) > loss(sham)					
brain structure	*P*	*x*	*y*	*z*	*T/K*
ROI					
L putamen	0.011	−28	−12	−2	4.98
**loss(theta) > loss(sham)**					
WB cluster level					
R calcarine cortex	<0.001	4	−68	14	12802
L postcentral gyrus	<0.001	−52	−12	40	664
R pallidum	<0.001	20	0	−10	349
L supplemental motor cortex	0.033	−8	4	60	100
L putamen	<0.001	−24	4	4	1414
L anterior cingulate cortex	0.006	12	44	8	159
L inferior frontal gyrus, opercular part	0.003	−46	22	8	189
R middle frontal gyrus	0.011	26	2	48	138
R fusiform gyrus	0.014	32	−48	−30	128
R thalamus	0.015	8	−14	0	123
ROI					
L amygdala	0.016	−24	0	−12	4.01
R amygdala	0.001	20	0	−12	5.30
R caudate	0.015	16	16	0	4.78
R cingulum anterior	0.001	12	44	8	6.26
L DLPFC (BA9)	0.012	−32	20	36	4.48
R vmPFC (BA10)	0.002	12	46	8	5.43
L putamen	0.001	−24	4	4	6.28
R putamen	0.003	22	6	−8	5.69

All coordinates (x, y, z) are given in MNI space.

*WB* whole brain, *L* left, *R* right.

**Table 4:** Localization and statistics of the voxels for the contrast loss (theta) > loss (beta).

brain structure	*P*	*x*	*y*	*z*	*K*_*E*_
WB cluster level					
R cerebellum	<0.001	30	−68	−34	17146
R caudate	0.001	16	16	2	665
L cerebellum	<0.001	−24	16	18	1255
L pallidum	<0.001	−20	−4	−6	307
R middle temporal gyrus	0.001	58	−30	−6	214
R precentral gyrus	0.002	52	6	34	166

All coordinates (x, y, z) are given in MNI space.

*WB* whole brain, *L* left, *R* right.

**Fig. 4:**
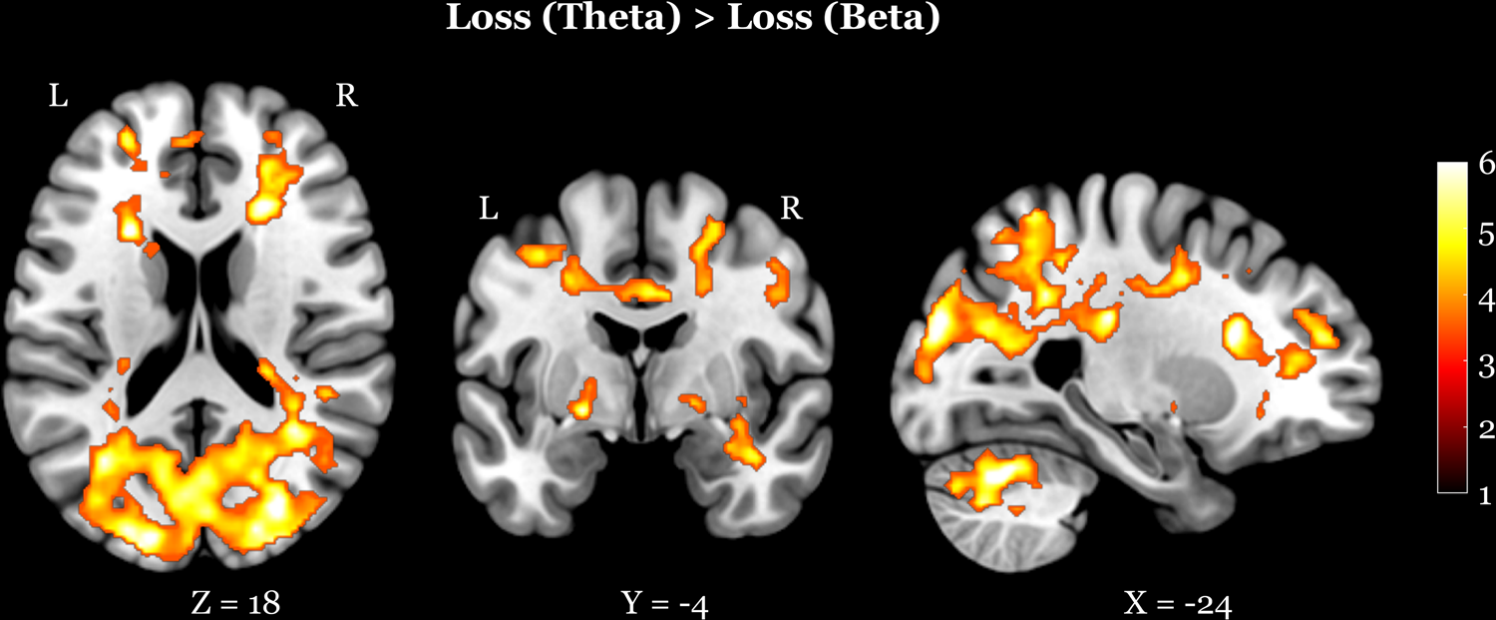
Areas showing activations during loss feedback processing for the contrast theta > beta. The opposite contrast, beta > theta, did not reveal significant results.

The analyses comparing beta and theta tACS revealed significant activations for the loss (theta) > loss (beta) contrast (Fig. 4, Table 4) in bilateral cerebellum (R cerebellum *p* < 0.001, x = 30, y = −68, z = −34, K_E_ = 17146; L cerebellum *p* < 0.001, x = −24, y = 16, z = 18, K_E_ = 1255), right caudate nucleus (*p* < 0.001, x = 16, y = 16, z = 2, K_E_ = 665), left pallidum (*p* < 0.001, x = −20, y = −4, z = −6, K_E_ = 307), right muddle temporal gyrus (*p* = 0.001, x = 58, y = −30, z = −6, K_E_ = 214) and right precentral gyrus (*p* = 0.002, x = 52, y = 6, z = 34, K_E_ = 166). No significant effects were found for the reverse loss contrast (loss (beta) > loss (theta)) or for gain-related contrasts.

### Relationship between BOLD and impulsive personality

**Fig. 5:**
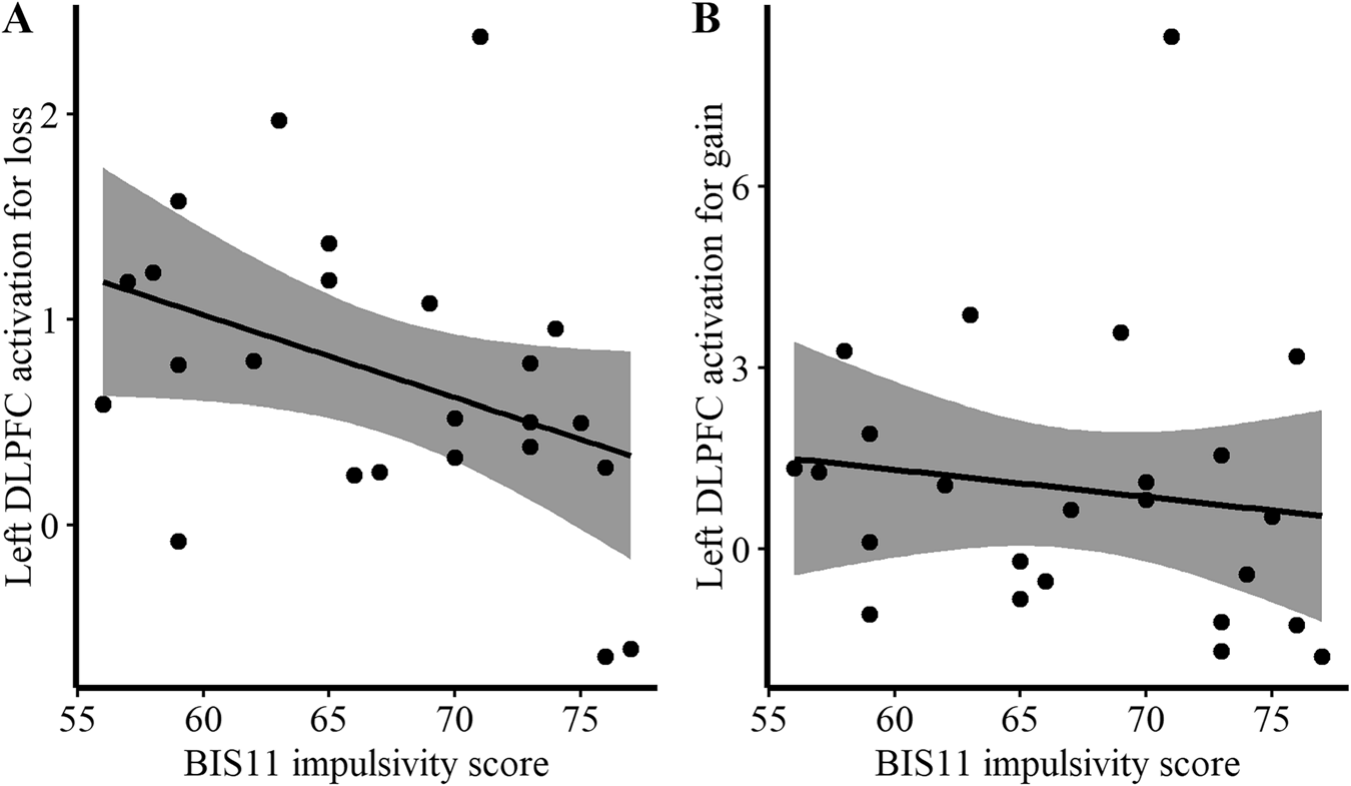
Scatterplots showing the relationship between BOLD activations and impulsivity in the theta stimulation condition. 5A shows the associations between impulsivity and left DLPFC activations during negative (loss) feedback, and 5B shows the relationship during positive (gain) feedback processing.

In the theta stimulation condition, impulsivity (BIS-11 total) was significantly negatively associated with left DLPFC activation for the loss contrast (theta > sham; *r* = −0.455, *p* = 0.025), and showed no significant correlation for the gain contrast (theta > sham; *r* = −0.332, *p* = 0.111). These results suggest that higher impulsivity is associated with reduced left DLPFC activation during loss feedback processing under theta stimulation. Figure 5 shows the correlations between brain activity and impulsivity measures.

## Discussion

This study investigated how theta (5 Hz) and beta (25 Hz) tACS modulated brain activity during reward-related feedback processing in healthy individuals. Participants performed a gambling task while receiving sham, theta, or beta tACS over the left DLPFC. We recorded fMRI simultaneously with tACS. Theta stimulation increased activation in the DLPFC, calcarine cortex, anterior cingulate cortex, inferior frontal gyrus, sensory-motor areas, amygdala, and basal ganglia regions during loss feedback. These regions have been implicated in sensory processing, error monitoring, cognitive control, and emotion regulation [47–50]. In contrast, beta stimulation primarily increased activation in the putamen and amygdala during gain feedback, regions linked to reward sensitivity and affective processing [51, 52]. Moreover, impulsivity was associated with stimulation-induced activity in key components of the feedback processing network. Specifically, higher impulsivity was associated with reduced DLPFC activation during loss processing in the theta condition. Our findings suggest that frequency-specific neuromodulation can influence how the brain processes feedback, and individual characteristics may affect the brain’s response.

During loss feedback, the theta-tACS modulated activation across broader regions involved in sensory, affective, and cognitive processes. Activation was significantly increased in the calcarine cortex and occipital areas, reflecting enhanced cortical synchronization that underlies heightened visual attention to loss-related feedback [47]. Increased activity in sensory-motor areas, such as the postcentral and precentral gyri, likely reflects sensorimotor integration that contributes to adaptive behavioural responses [48]. Furthermore, the left anterior cingulate cortex and left inferior frontal gyrus were activated, which are involved in error monitoring, cognitive control, and emotional regulation during decision-making [49, 50]. Enhanced activation in the amygdala and basal ganglia regions, such as the putamen and caudate, points to the affective processing in response to negative outcomes. Affective processes may influence cortico-basal ganglia circuits, which integrate learning and emotion to guide the selection and evaluation of actions [53]. Increased activation in the DLPFC, a region associated with executive functions, cognitive control, and decision-making [54], further supports the involvement of higher-order cognitive processes in regulating emotional responses to loss. We found that beta tACS significantly increased activation in the putamen bilaterally and in the left amygdala during gain feedback. The putamen, a critical component of the striatum, plays a key role in integrating reward signals and guiding action selection [3, 53]. The increased activity in this region suggests enhanced engagement of basal ganglia circuits during the processing of positive feedback. Additionally, the observed activation in the left amygdala, a crucial component of the mesolimbic circuitry [51, 52], suggests that beta tACS might influence affective components of reward processing.

Our results suggest that theta and beta tACS modulate distinct neural networks during the processing of loss and gain feedback. Theta tACS enhances activation in brain regions critical for error monitoring, cognitive control, and emotion regulation, particularly during negative feedback. In contrast, beta tACS influences activation in areas involved in integrating affective and reward-related information during positive feedback. Our findings are consistent with the previous studies from our group and others on feedback processing that have identified distinct neural networks for gain and loss feedback. In a prior EEG-fMRI study on healthy subjects, our group found that theta oscillations correlated with ACC and mPFC activity during negative feedback, while beta oscillations were associated with ventral striatum activity during gain feedback processing [18]. Kringelbach and Rolls [50] argued that reward and punishment have spatially distinct representations in the human brain. The ventral striatum and orbitofrontal cortex are involved in reward or positive feedback, whereas the ACC and amygdala play a key role in processing negative feedback [50]. Overall, our findings reinforce that feedback processing is mediated by distinct yet interconnected brain regions, and theta and beta oscillations play complementary roles in engaging these regions.

Our findings demonstrated that tACS selectively enhanced BOLD activations in brain regions associated with processing gain and loss feedback. Although the exact neuronal mechanism by which tACS modulates BOLD activation remains unclear, current research suggests that this modulation may be driven by neuronal entrainment. According to the entrainment hypothesis, continuous stimulation with oscillating current causes neuronal populations to synchronize their activity with the externally applied rhythm, thereby modulating neuronal firing patterns and large-scale functional connectivity [55, 56]. Neurons whose intrinsic frequencies align with the stimulation frequency are more likely to be entrained, whereas those firing at different frequencies remain unaffected [55, 56]. In this study, tACS over left DLPFC may have enhanced synchrony between cognitive control regions and the feedback processing network. This network-level synchronization could manifest as changes in the BOLD signal. Simultaneous tACS-fMRI studies in other cognitive domains provide further support for frequency-specific entrainment as a key mechanism underlying the effects of tACS [57, 58]. Vosskuhl et al. [57] reported frequency-specific BOLD modulation during simultaneous alpha tACS-fMRI and argued that tACS modulated BOLD activation via oscillatory entrainment across large-scale networks. Similarly, Kar et al. [58] found that tACS increased functional connectivity of the stimulated human motion area (hMT+) with the widespread cortical regions and the dorsal attention network in particular. Together, these findings reinforce the idea that weak oscillatory currents can synchronize neural populations and influence both local and distributed brain activity through frequency-dependent entrainment of neural networks.

Left DLPFC activity was associated with impulsive personality traits. Specifically, higher impulsivity was negatively correlated with left DLPFC activation during loss feedback in the theta stimulation condition. These findings are consistent with previous studies reporting a negative correlation between impulsivity scores and theta power in response to negative feedback [13, 40]. Moreover, a previous fMRI study in patients with impulsivity-related personality disorders found reduced prefrontal activation during both reward and loss compared to healthy controls [59]. Our results suggest that reward-related feedback processing deficiency might underlie impulsive personality. Alternatively, the observed reduction in theta-related DLPFC activation in high-impulsivity individuals could reflect a deficit in cognitive control processes that depend on the feedback to guide behavior.

Some possible limitations of our study should be noted. Our stimulator comprised one stimulating electrode and one return electrode, which may limit the precise distribution of current over the target area. Another limitation is that the simple gambling paradigm used in this study did not allow monitoring of participants’ expectations, making it difficult to differentiate the effects of reward and loss from those of prediction error. Finally, although tACS modulated BOLD activity in the feedback network, these neural effects did not translate into measurable behavioral changes, as participants’ risk-taking behavior did not differ across stimulation conditions. Future studies could employ additional behavioral measures or more sensitive tasks to better link tACS-induced neural modulation to behavior.

In summary, our findings demonstrate that the application of tACS at theta and beta frequencies over the left DLPFC preferentially modulates brain activity underlying the processing of positive and negative feedback. Theta tACS primarily modulated activation in brain regions involved in error monitoring, cognitive control, and adaptive regulation during loss feedback, whereas beta tACS influenced activation in areas involved in emotion regulation and reward-guided decision-making during gain feedback. This differentiation in neuronal modulation provides further evidence of the unique roles that different brain oscillations play in regulating feedback processing. Neuromodulation is potentially achieved through the entrainment of neural oscillations and the enhancement of network-wide synchronization. These findings contribute to our understanding of the neural mechanisms involved in feedback processing and may support future research on neuromodulatory interventions in psychopathological conditions.

## Data Availability

Data supporting the results of this study are available from the corresponding author upon request.
